# Sternal Elevation by Crane Technique During Double Lung Transplant for Patient With Pectus Excavatum

**DOI:** 10.1016/j.atssr.2024.02.001

**Published:** 2024-03-02

**Authors:** Arizona Binst, Yanina Jansen, Laurens J. Ceulemans, Dirk Van Raemdonck, Hans Van Veer

**Affiliations:** 1 Department of Thoracic Surgery, University Hospital Leuven, Leuven, Belgium; 2 BREATHE Laboratory, Department of CHROMETA, KU Leuven, Leuven, Belgium

## Abstract

We present the case of a 28-year-old female patient who underwent a bilateral lung transplantation for underlying terminal bronchopulmonary dysplasia. The peroperative access to the hilum of the right lung was significantly compromised due to the presence of a pectus excavatum (Haller index 11). We used a wired sternal crane technique to elevate the sternum and gain exposure. Release of the crane after implantation went smoothly, as did the postoperative recovery. This report illustrates the feasibility of this technique during lung transplantation.

Pectus excavatum encompasses an inward depression of the sternum. This can cause cardiopulmonary restriction when the depression causes a deformation of the heart, decreasing its expansion. The right ventricle is predominantly affected. The most common surgical correction nowadays is the thoracoscopic placement of one or more Nuss bars.^1^

The presence of a pectus excavatum becomes a more profound problem in patients with pulmonary disease necessitating thoracic surgery. The severe pectus excavatum in our patient confronted us with a particular exposure challenge during bilateral lung transplantation.

Informed consent for this case report and use of images was obtained directly from the patient and her legal representatives. Consent of the local ethics committee was obtained (reference number S67600).

We present the case of a 28-year-old woman who suffered from terminal bronchopulmonary dysplasia for which she underwent a double lung transplant at our institution in the course of 2022. The patient had a deep pectus excavatum with a Haller index of 11 and correction index of 48% (Figure 1, Figure 2). Initial repair of the chest wall depression was not performed as her clinical condition did not allow staged approach and the Nuss bars would hinder bilateral anterior thoracotomy. Appropriate donor organs of a 64-year-old nonsmoker deceased of cardiac death donor were offered. Following our institutional technique of clamshell-avoiding sequential single-lung transplantation, a right anterior thoracotomy was performed with access in the 4th intercostal space. After opening the chest, the pectus excavatum compromised exposure of the hilum and access to the mediastinal part of the main right pulmonary artery. Subsequently, a symmetrical anterior thoracotomy on the left side was performed revealing several adhesions and a fibrothorax. Then, a double threaded steel wire no. 5 was passed behind the sternum and a pectus crane (Pectus Crane System retractor, Thompson Surgical Instruments) was used to lift the sternum (Figure 3). An additional 4 cm in anteroposterior distance was achieved, leading to a significantly increased exposure of the right pulmonary artery. The crane position remained unchanged throughout the procedure. After implantation of the first, right lung, increased pulmonary artery pressure due to “first-lung syndrome”′ necessitated peripheral venoarterial extracorporeal membrane oxygenation (ECMO) support. Central ECMO was not possible due to the pectus also decreasing visualization and space towards the aortic arch. After implantation of both lungs, the patient could be weaned from ECMO without impact on cardiovascular status. Lowering of the sternum caused no changes in hemodynamics nor ventilation. A definitive pectus-correction was not performed.

**Figure 1:**
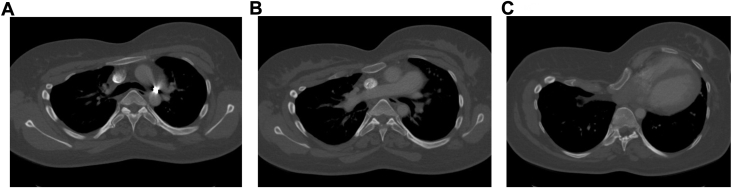
Contrast-enhanced computed tomography scan of the chest with moderate asymmetrical pectus excavatum and platythorax type chest. (A) Axial image of the sternum at the level of the carina. (B) Axial image at the level of the truncus of the pulmonary artery showing a rotation of the sternum. (C) Axial image at the deepest point of the pectus excavatum at the level of the ventricles.

**Figure 2:**
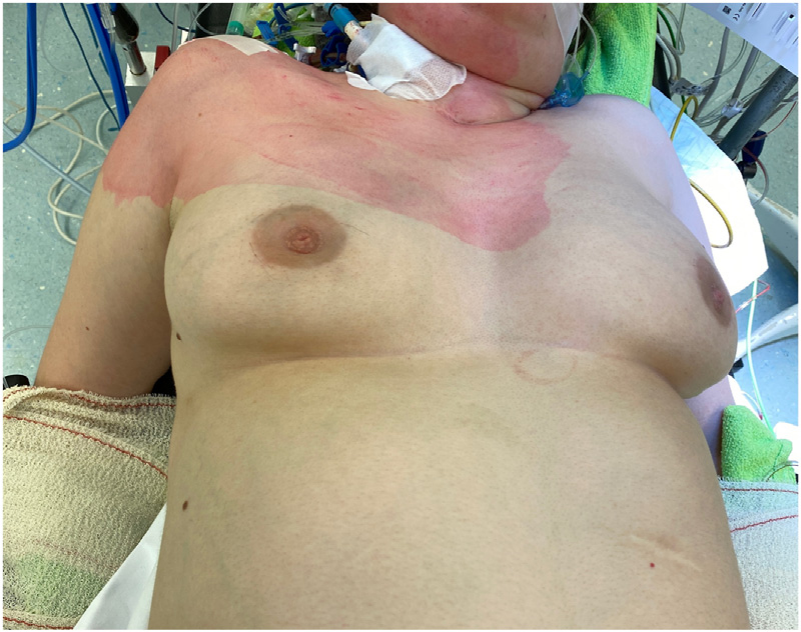
Clinical picture of the pectus excavatum, before start of transplantation.

**Figure 3:**
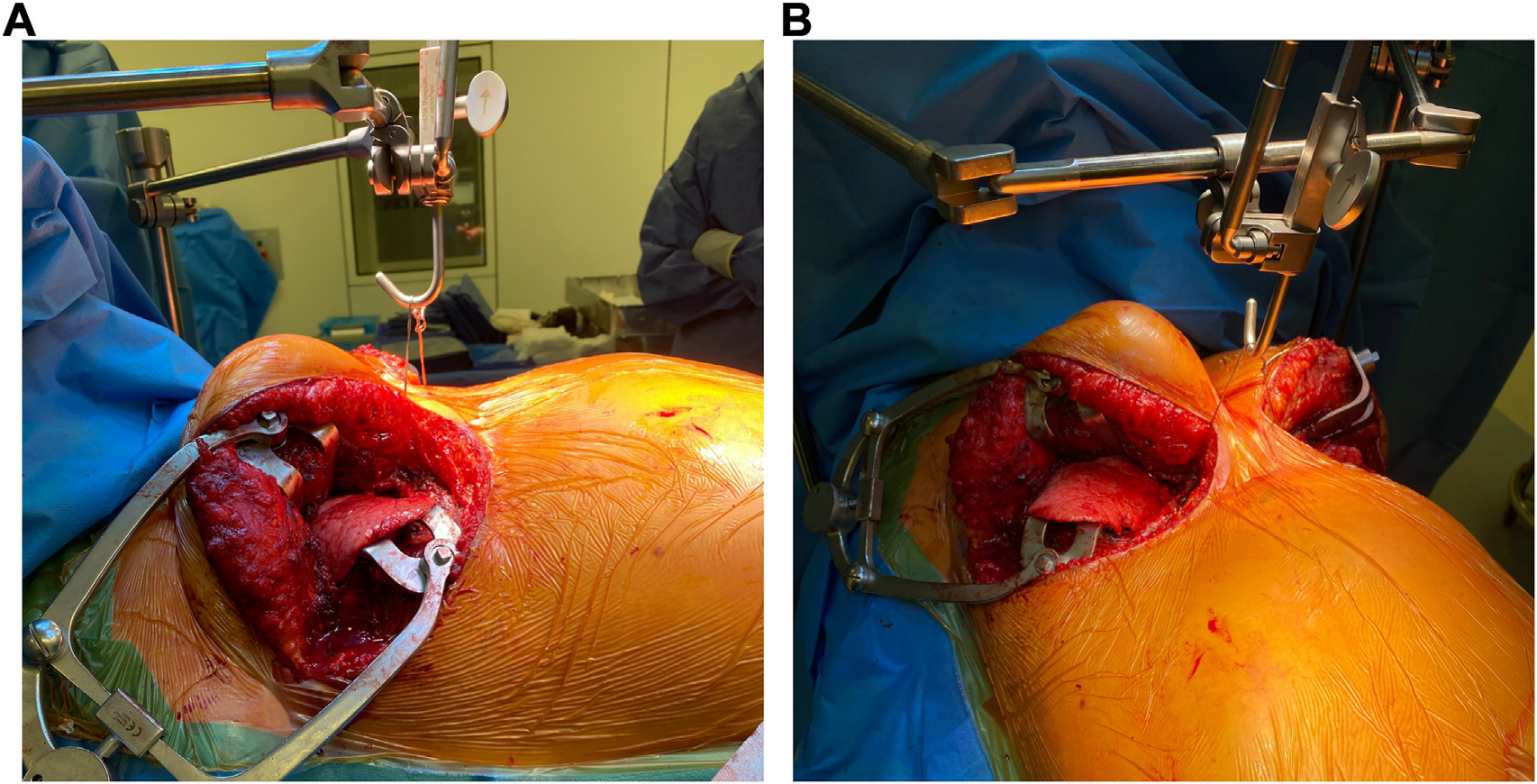
(A) Lateral view of pectus crane installation during surgery. (B) Anterior view of pectus crane installation during surgery.

Total duration of the procedure was 10 hours 46 minutes with an anastomosis time of 106 minutes on the right side and 86 minutes on the left. Total ischemia time of the lungs was, respectively, 522 and 768 minutes. Total time on venoarterial ECMO was 170 minutes.

The prolonged anastomosis time was related to the technically challenging anastomoses on the pulmonary arteries due to important anatomical donor to recipient mismatch. We believe that the exposure in this thorax with uncorrected pectus deformity—in itself—had no influence on the total ischemia time, as we had corrected this issue well before starting the anastomoses by use of the crane. Other contributing factors were the placement of the ECMO-circuit and logistics issues (short period of initial unavailability of the operating room). There was no major bleeding.

Postoperatively, the patient suffered an anaphylactic shock to recombinant thymoglobulin. Further postoperative course was uneventful. Primary graft dysfunction scores at 24, 48, and 72 hours were 3, 2, and 2, respectively. There was a small dehiscence of the left incision for which a wound debridement and primary closure was performed. The patient could be discharged home on day 27 with classic triple immunosuppression.

## Comment

The sternal crane technique has been proven to provide a safe and useful way for forced sternal elevation and mediastinal passage during pectus excavatum repairs.^1^ In this case, we used the crane to elevate a sternum during a bilateral lung transplantation, allowing temporary access to the hilum and thus avoiding the need for a clamshell thoracotomy. In our center, this clamshell-avoiding approach is the preferred method of action, given the increased risk of partial right ventricular compression by the distal half of the sternum and risk of complicated postoperative osseous healing.

Previous case reports have described the implantation of Nuss bars concomitant to bilateral lung transplantation.^2^, ^3^, ^4^ We did not correct the chest wall deformity at listing for transplantation because the patient’s pulmonary condition was too limited. Moreover, the eventual Nuss bars would be at the exact level where the bilateral anterior thoracotomies need to be performed, causing vast adhesions of the recipient lungs. The Nuss procedure was also not performed at transplantation, due to concerns for postoperative infections, bleeding, and impaired wound healing. In fact, a wound debridement was necessary just before hospital discharge, which could have been a potential hazard for material infection.

The pectus correction being performed only after implantation of the donor lungs, no improved visibility on the mediastinal access during transplantation could be expected.

Similarly, considering a Ravitch procedure before implantation to improve mediastinal access would increase ischemia time and blood loss, together with a risk of potential infection of the implants used to maintain the position of the sternum.

Currently, the presence of significant chest wall or spinal deformity is a relative contraindication for lung transplantation according to the International Society for Heart and Lung Transplantation guidelines,^5^ due to technical difficulties it may cause during surgery and the concern for postoperative respiratory complications. We suggest that the use of the pectus crane should be considered to improve the intraoperative surgical exposure.

Additionally, we suggest that transplant candidates with chest wall deformities be discussed with experienced chest wall surgeons, before listing, to evaluate the possibility of a staged approach.

In conclusion, the sternal crane provided us with an easy and safe way to gain additional anteroposterior exposure during lung transplant avoiding clamshell thoracotomy. The pectus crane may also be used in other types of thoracic surgery to provide additional space in the chest when deemed necessary.
